# Barriers to Buprenorphine Initiation in Patients Using Fentanyl

**DOI:** 10.1001/jamanetworkopen.2025.52136

**Published:** 2026-01-05

**Authors:** Sarah S. Kawasaki, Jane M. Liebschutz, Cristina Murray-Krezan, Galen E. Switzer, Samantha Nash, Kwonho Jeong, Erin L. Winstanley

**Affiliations:** 1 Penn State Milton S. Hershey Medical Center, Hershey, Pennsylvania; 2 Division of General Internal Medicine, Center for Research on Health Care, University of Pittsburgh, Pittsburgh, Pennsylvania; 3 University of Pittsburgh Medical Center, Pittsburgh, Pennsylvania; 4 Center for Biostatistics and Qualitative Methodology, Division of General Internal Medicine, University of Pittsburgh, Pittsburgh, Pennsylvania

## Abstract

**Importance:**

Anecdotal accounts suggest an increase in problems initiating buprenorphine (BUP) treatment among individuals using illicitly manufactured fentanyl. Limited empirical data illuminate these challenges.

**Objective:**

To determine the prevalence of clinician-reported problems initiating BUP treatment among patients using fentanyl and describe clinical strategies used to overcome engagement challenges.

**Design, Setting, and Participants:**

For this survey study, an online survey was pilot tested and refined with a convenience sample of physicians. The final survey included 96 items and took less than 15 minutes to complete. The survey queried patients’ use of fentanyl, BUP induction problems (precipitated or prolonged withdrawal), strategies to overcome induction problems, clinician characteristics, and practice characteristics. Eligible clinicians initiated BUP for at least 10 patients with opioid use disorder in the past year and at least 1 patient in the past 90 days. The survey was live from June 2, 2023, to March 18, 2024.

**Main Outcome and Measures:**

The main outcome of interest was precipitated and/or prolonged opioid withdrawal. Descriptive statistics are reported, and logistic regression was used to identify factors associated with BUP initiation problems.

**Results:**

A random sample of physicians and advanced practice clinicians in the US Drug Enforcement Administration (DEA) registrant dataset from October 2022 (n = 3141) were invited to participate; of 2485 eligible for inclusion, 649 (26.1%) completed the prescreen survey. Of 421 (64.9%) eligible to complete the survey, the final sample included 396 (94.1%) clinicians who completed at least 50% of the survey items. Of 390 participants, 284 (72.8%) reported problems when initiating BUP in patients using fentanyl, with 242 of 394 (61.4%) reporting patients’ experiencing precipitated withdrawal. A total of 264 or 392 participants (67.3%) reported modifying their standard induction procedures, changing how they counsel patients, or changing both medication and counseling protocols. In multivariable modeling, clinicians were more likely to report problems initiating BUP in patients if they had a DEA waiver to treat more than 100 patients (OR, 1.92; 95% CI, 1.08-3.40), vs those waivered to treat fewer patients; if they reported at least 75% of their patients using fentanyl (OR, 6.31; 95% CI, 2.59-15.35), vs no patients; or if they inducted patients in noninpatient settings (OR, 2.79; 95% CI, 1.39-5.61), vs inpatient settings.

**Conclusions and Relevance:**

In this survey study of clinician-reported problems initiating BUP treatment, clinicians working in high-volume noninpatient settings reported more problems initiating BUP in patients using fentanyl, and many reported changing their clinical practices in response to these problems. Further research is warranted to match alternate BUP induction strategies by clinical settings.

## Key Points

**Question:**

How frequently do clinicians encounter problems initiating buprenorphine among patients using fentanyl, and how are their clinical practices changing?

**Findings:**

In this survey study, the majority of 396 clinicians surveyed (72.0%) reported encountering problems initiating buprenorphine treatment among patients using fentanyl in the past year, and 67.3% reported modifying their standard induction protocols.

**Meaning:**

These findings suggest that clinicians across the US are experiencing challenges initiating buprenorphine treatment, and many may be changing their clinical practices to circumvent these problems.

## Teaser

This survey study examines the prevalence of clinician-reported problems related to initiating buprenorphine treatment for individuals using fentanyl and explores how these problems are changing clinical practice.

## Introduction

Buprenorphine (BUP) treatment reduces illicit opioid use and opioid-related overdose deaths^1,2^; however, suboptimal access and engagement in treatment programs offering medications for opioid use disorder undermines public health efforts to reduce overdose deaths. Since 2016, opioid overdose deaths have increasingly involved illicitly manufactured fentanyl (IMF).^3,4,5^ Simultaneously, BUP prescribers across the US have anecdotally reported problems initiating treatment, while others have reported that some patients using IMF are unwilling to engage in BUP treatment.^6^ Fentanyl, given its lipophilicity, has a variable clearance rate ranging from a few days to weeks.^7,8^ Studies have documented problems, primarily precipitated opioid withdrawal, during BUP induction in individuals using IMF,^9,10,11^ and that patients using IMF reported that BUP does not work for them.^10,12^ Self-reported data collected from various substance use disorder (SUD) treatment centers across the US found that BUP-mediated precipitated withdrawal among those using IMF was more than 5 times higher than for those using other opioids (OR, 5.202; 95% CI, 1.979-13.675).^13^ The half-life of the opioids used and time of last use influences the time course and severity of opioid withdrawal, wherein longer-acting opioids may cause prolonged withdrawal.^14^ For fentanyl, secondary peaking or fentanyl rebound and the long terminal elimination may contribute to prolonged withdrawal.^15^ Standard BUP induction procedures may not work for individuals using IMF, and in response, clinicians are adopting alternative strategies during BUP induction or referring patients to available methadone programs.^16^ Alternative initiation strategies in the literature have included low-dose or micro-inductions, starting with less than 1 mg of BUP, designed to limit withdrawal symptoms by using a cross taper between BUP and full agonist opioid.^17^ High-dose initiation (macro-inductions) initial doses range from 16 mg to 64 mg.^18^ Direct-to-inject protocols involve long-acting injectable BUP, which has a gradual increase in BUP level.^18^

The national penetration of IMF into the drug supply and its associated problems with BUP-precipitated withdrawal are inadequately studied. One study reported that 19.6% of patients entering addiction treatment reported using IMF.^19^ A recent multisite clinical trial conducted in emergency departments (EDs) reported that only 0.76% of participants (n = 9) testing positive for IMF experienced precipitated withdrawal, defined as a score of 5 or higher on the Clinical Opioid Withdrawal Scale (COWS) instrument, with initiation of injectable BUP,^20^ whereas a recent retrospective analysis found a 10-fold higher incidence of precipitated withdrawal among patients using IMF who initiated BUP in the ED, with different doses in each study.^21^ Research suggests that IMF is more widely available east of the Mississippi River,^22^ but there is evidence that IMF has penetrated West Coast markets.^23^ In sum, the mixed results from existing studies make it difficult to determine whether BUP induction problems in patients using IMF are widely problematic, are located in isolated geographic regions, or are more acute in particular clinical settings.

Prior to the widespread availability of IMF, approximately 27% of patients did not complete the BUP induction phase.^24^ Standard clinical practice, developed before the penetration of IMF into the drug supply, requires patients to abstain from short-acting opioids for 12 hours and long-acting opioids for 30 to 48 hours prior to initiating BUP.^25^ Within the past 5 years, some patients reported experiencing precipitated withdrawal even after abstaining for 24 to 48 hours.^13,26^ Alternative low-dose and high-dose BUP induction protocols have been developed and tested in small single-site studies.^27,28^ The precipitated withdrawal rates published in professional society guidelines^14^ and in conflicting reports in the ED setting^21,23^ ranged from minimal to 10-fold higher than previously reported. The purpose of this study was to estimate the national prevalence of clinician-reported problems initiating BUP and summarize how these problems are changing clinical practices. We hypothesized that more than 40% of clinician respondents would report problems initiating BUP treatment.

## Methods

The development and reporting of this survey comply with the American Association for Public Opinion Research (AAPOR) reporting guideline.^29^ The study was approved by the institutional review boards at the University of Pittsburgh and the Pennsylvania State University College of Medicine. Participants provided written consent at the start of the survey instrument before being permitted to complete the survey.

### Survey Development

A team of addiction medicine physicians, content area experts, and survey development experts created a survey to measure problems initiating BUP treatment in patients, because no psychometrically validated instruments exist. The initial survey draft was pilot-tested internally and reviewed by the study team; a revised survey draft was then pilot-tested with a convenience sample (n = 12) of addiction medicine physicians who prescribe BUP. The sample included at least 1 clinician from each of the 10 Department of Health and Human Services regions^30^ and from different clinical settings (eg, ED, hospital, outpatient SUD, opioid treatment program). Clinicians reviewed the web-based survey and provided written feedback on individual survey items. The study team conducted brief video conference calls with clinicians to solicit a more in-depth critique of the survey and to ensure that the study team understood their recommendations. The team then revised it to create the final survey, which took less than 15 minutes to complete.

The survey included 96 questions across 5 domains: (1) characteristics of patients using IMF (18 items), (2) standard care for BUP induction and problems with BUP initiation (18 items), (3) strategies used to reduce problems associated with BUP induction (15 items), (4) clinician characteristics (21 items), and (5) practice characteristics (24 items). In the clinician and practice sections of the survey, we integrated some questions from validated surveys.^31,32^ To reduce recall bias, problems initiating BUP were framed within the past year. Additionally, we asked about patients’ preferences for methadone, desire to discontinue BUP, and need for concomitant medications during BUP induction.

### Survey Sample

A stratified random sample of X**-**waivered clinicians was selected using the US Drug Enforcement Administration (DEA) registrant database (obtained on September 28, 2022, prior to removal of the DEA X-waiver requirement), and clinicians practicing in US territories were excluded. The sample strata included census regions (Northeast, Midwest, South, West), prescriber type (physician vs nurse practitioner or physician assistant), and number of patients waivered to treat (<31, 31-100, 101-275). The study team searched online to verify the status of selected prescribers’ medical licenses and to extract contact information, including email addresses. The study team also called prescribers to verify practice and contact information as needed. Nonresponses and reasons for refusal were tracked.

We distributed the survey in 2 waves. Wave 1 included 922 clinicians, and wave 2 included 2219 clinicians. We evaluated the sampling distribution of the wave 1 respondents to improve strata balance and restricted wave 2 to clinicians waivered for at least 100 patients, and we oversampled clinicians in the Northeast.

### Survey Administration

We first mailed prenotification postcards to selected clinicians.^33^ We emailed or mailed the survey invitation letter 5 to 7 days later and sent a reminder postcard another 5 to 7 days later. Study staff attempted telephone contact with survey nonresponders. The survey was live from June 2, 2023, to March 18, 2024.

### Inclusion and Exclusion Criteria

Clinicians completed a brief web-based screen to determine eligibility. Eligibility criteria included (1) having an active X-waiver in October 2022, (2) having initiated BUP treatment with at least 10 patients in the past year, and (3) having initiated BUP treatment with at least 1 patient in the past 90 days. Per protocol, we excluded participants who completed less than 50% of the survey.

### Outcome Measures

The primary outcome was the percentage of participants reporting any problems in the past year with initiating BUP in patients using IMF due to precipitated withdrawal and/or prolonged withdrawal. The survey defined precipitated withdrawal as an initial increase of at least 10 points on the COWS scale or a rapid onset of new withdrawal symptoms after BUP administration^34^ and prolonged withdrawal as withdrawal symptoms lasting at least 24 hours.^35^ If participants answered yes to either question, they were categorized as having BUP induction problems. Secondary outcomes were (1) the proportion of participants who modified their standard BUP induction procedure for patients using IMF, (2) the proportion of participants who reported lower rates of BUP retention among patients using IMF, and (3) the proportion of participants who had referred patients to methadone treatment.

Covariates in the multivariable analysis included census regions (Northeast, Midwest, South, and West); clinician type (physician, physician assistant, and nurse practitioner); X-waiver patient limits (30, 100, and 275); practice setting (outpatient only, inpatient only, ED only, other, and a combination of settings) and BUP induction settings (only outpatient, only inpatient, and combined). Practice settings capture treatment settings in which the participants work, whereas BUP induction settings are where the patient is at the time of induction. Other covariates included in multivariable analyses were the proportion of patients using IMF and number of years the clinician was in practice. Information on clinicians’ race and ethnicity was collected to identify descriptive statistics and ensure there were no significant differences among clinicians sampled. Respondents reported their own race and ethnicity from a list of provided options (American Indian or Alaska Native, Asian or Pacific Islander, Black, White, multiracial, or some other race; and Hispanic or non-Hispanic ethnicity).

### Statistical Analysis

We calculated medians and IQRs for continuous variables and frequencies and percentages for categorical variables to summarize the survey responses. We assessed bivariable associations between outcomes and covariates and identified variables that needed recoding for modeling due to small cell counts. For example, because of small cell counts, we created a composite variable for clinician type and number waivered. We used multivariable logistic regression modeling to determine whether any covariates were associated with problems initiating BUP treatment. Adjusted odd ratios and their 95% CIs were estimated, and we report results from the parsimonious model that included the stratification variables. *P *values were calculated using Pearson χ^2^ or from a Fisher exact test where indicated, and a 2-sided *P* < .05 indicated statistical significance. Analyses were performed in Stata, version 18 (StataCorps LLC).^36^

## Results

### Sample Characteristics

Of 3141 participants randomly selected from the DEA database, 76 were excluded prior to survey distribution due to having an inactive or revoked license or being deceased, and 580 did not have confirmable contact information, leaving 2485 (Figure). Of these, 649 (26.1%) participants completed screening for the survey and 421 (64.9%) were eligible. Reasons for ineligibility (n = 228) included the following: The clinician conducted less than 10 BUP inductions in the last year (n = 180), conducted less than 1 BUP induction in the last 90 days (n = 24), opted out of the survey (n = 16), or did not have or use a BUP waiver (n = 6); or the incorrect person completed the survey (n = 2). Among the 421 clinicians eligible for the study, 405 initiated the survey. Nine participants were excluded for completing less than 50% of the survey, yielding a final analytical sample size of 396 (94.1% response rate).

**Figure.**
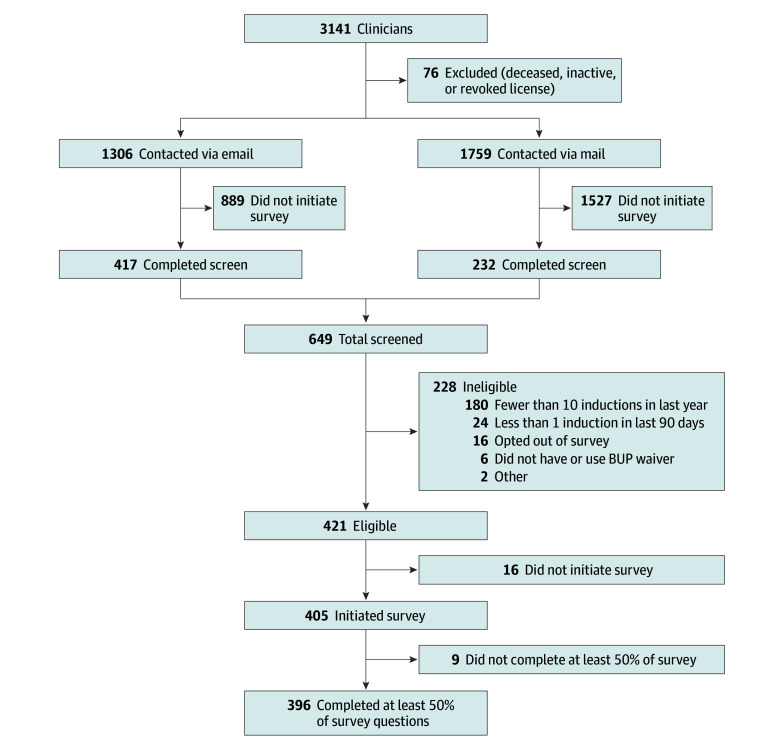
Study Flow Chart

### Sample Characteristics and Practice Settings

Of the 393 participants who reported gender, 203 (51.7%) were women, 184 (46.8%) were men, and 6 (1.5%) reported other gender or preferred not to answer. Of 390 participants who reported race and ethnicity, 3 (0.77%) were American Indian or Alaska Native; 52 (13.3%) were Asian or Pacific Islander; 291 (74.6%) were White; 21 (5.4%) were multiracial or some other race; 6.7% were Hispanic; and 364 (93.3%) were non-Hispanic. The median age was 45 years (range, 25-88 years; IQR, 27-63 years). Participants had been practicing medicine for a median of 6 years (range, 1-48 years; IQR, 0-13 years) (Table 1). The regional representativeness of the sample was nearly identical to the distribution in the DEA database.

**Table 1.** Demographic and Practice Characteristics of Clinicians Responding to Survey

Variable	Total sample, No. (%) (N = 396)	Reported ≥1 case of precipitated or prolonged withdrawal, No. (%) (n = 379)		*P* value
		No	Yes	
Age, y				
No. of respondents	389	93	279	
<34	51 (13.1)	9 (9.7)	40 (14.3)	.16^a^
35-44	132 (33.9)	33 (35.5)	94 (33.7)	
45-54	97 (24.9)	19 (20.4)	74 (26.5)	
55-64	67 (17.2)	16 (17.2)	46 (16.5)	
>65	42 (10.8)	16 (17.2)	25 (9.0)	
Gender identity				
No. of respondents	393	94	282	
Man	184 (46.8)	44 (46.8)	131 (46.5)	.52^b^
Woman	203 (51.7)	48 (51.1)	147 (52.1)	
Other or prefer not to answer	6 (1.5)	2 (2.1)	4 (1.4)	
Ethnicity				
No. of respondents	390	94	279	
Hispanic	26 (6.7)	4 (4.3)	22 (7.9)	.23^a^
Non-Hispanic	364 (93.3)	90 (95.7)	257 (92.1)	
Race				
No. of respondents	390	93	280	
American Indian or Alaska Native	3 (0.77)	2 (2.2)	1 (0.4)	.37^b^
Asian or Pacific Islander	52 (13.3)	13 (14.0)	36 (12.9)	
Black	23 (5.9)	5 (5.4)	16 (5.7)	
White	291 (74.6)	69 (74.2)	211 (75.4)	
Multiracial or some other race	21 (5.4)	4 (4.3)	16 (5.7)	
Census region				
No. of respondents	396	94	285	
Northeast	104 (26.3)	20 (21.3)	77 (27.0)	.44^a^
Midwest	72 (18.2)	18 (19.1)	50 (17.5)	
South	103 (26.0)	30 (31.9)	70 (24.6)	
West	117 (29.5)	26 (27.7)	88 (30.9)	
Clinician type				
No. of respondents	394		283	
MD	239 (60.7)	59 (62.8)	167 (59.0)	.17^a^
NP, PA, or other	155 (39.3)	35 (37.3)	116 (41.0)	
Years in practice treating patients for opioid use disorder				
No. of respondents	392	93	280	
<5	113 (28.8)	31 (33.3)	77 (28.8)	.51
5-10	180 (45.9)	37 (39.8)	135 (45.9)	
11-15	49 (12.5)	10 (10.8)	47 (12.5)	
16-19	22 (5.6)	7 (7.5)	22 (5.9)	
>20	28 (7.1)	8 (8.6)	26 (6.9)	
Primary practice setting				
No. of respondents	385	91	279	
Primary care practice	142 (36.9)	44 (48.4)	90 (32.3)	.09^a^
Mental health community treatment center	54 (14.0)	11 (12.1)	41 (14.7)	
Opioid treatment program	73 (19.0)	15 (16.5)	57 (20.4)	
Inpatient addiction treatment facility	21 (5.5)	1 (1.1)	19 (6.8)	
Hospital (inpatient)	28 (7.3)	8 (8.8)	20 (7.2)	
Emergency department	27 (7.0)	6 (6.6)	20 (7.2)	
Inpatient mental health facility	5 (1.3)	2 (2.2)	3 (1.1)	
Telehealth or remote	14 (3.6)	3 (3.3)	11 (3.9)	
Other	13 (3.4)	1 (1.1)	11 (3.9)	
Correctional facility (inpatient)	8 (2.1)	0	7 (2.5)	
In which settings do you induct patients on buprenorphine? (check all that apply)^c^				
No. of respondents	386	92	278	
Inpatient	122 (31.6)	23 (25.0)	96 (34.5)	NA
Outpatient clinic	289 (74.8)	73 (79.3)	207 (74.5)	
At home	113 (29.3)	16 (17.4)	93 (33.5)	
Other	17 (4.4)	5 (5.4)	12 (4.3)	
Transferred^d^	46 (11.9)	6 (6.5)	38 (13.7)	
Correctional facility	10 (2.6)	1 (1.1)	8 (2.9)	
ED	12 (3.1)	1 (1.1)	9 (3.2)	
Low barrier, street outreach, mobile	5 (1.3)	0	5 (1.8)	

Abbreviations: ED, emergency department; MD, medical doctor; NP, nurse practitioner; NA, not applicable; PA, physician assistant.

^a^ *P* value from Pearson χ^2^ test unless marked otherwise.

^b^ *P* value from Fisher exact test.

^c^ The number of responses is larger than the total number of respondents because the respondents were allowed to pick more than 1 answer. The percentages reported reflect the number of respondents checking the answer.

^d^ Transferred means that patients were inducted and titrated to an adequate dose in another setting and then transferred to the clinician for ongoing treatment with buprenorphine.

Most participants were physicians (239 of 394 [60.7%]) (Table 1). Participants reported a range of experience treating individuals with OUD: 113 of 392 (28.8%) reported less than 5 years; 180 (45.9%), between 5 and 10 years; and 99 (25.2%), for 11 years or longer. A total of 196 of 396 participants (49.5%) reported having a DEA waiver to prescribe BUP for less than 5 years. Of 386 respondents, 289 (74.9%) reported initiating BUP in outpatient settings for at least some of their practice, while 122 (31.6%) reported practicing in inpatient BUP induction settings as part of their total practice (addiction treatment, hospital, or mental health facility) (Table 1).

Of 394 respondents, 270 (68.5%) reported having patients with OUD constitute greater than one-quarter of their practice, and 156 of 385 (40.5%) reported working in 2 or more practice settings. Ninety of 396 respondents (22.7%) reported that patients receiving BUP were always observed in clinic taking their first dose and given prescriptions for at least 1 week at the first visit. Of 390 respondents, 277 (71.0%) reported becoming aware that patients were using IMF more than 1 year prior to the survey. Of 395 respondents, 263 (66.6%) reported routinely testing for fentanyl, and 69 or 257 (26.8%) had access to point-of-care fentanyl screening tests without confirmation (Table 2).

**Table 2.** Treating Patients With Opioid Use Disorder and Fentanyl Use

Variable	Total sample, No. (%) (N = 396)	Reported ≥1 case of precipitated or prolonged withdrawal, No. (%) (n = 379)		*P* value
		No	Yes	
Approximately what percentage of your patients have an OUD?				
No. of respondents	394	94	283	
<25	117 (29.7)	41 (43.6)	71 (25.1)	.008^a^
25-50	108 (27.4)	24 (25.5)	78 (27.6)	
51-76	68 (17.3)	11 (11.7)	55 (19.4)	
75-99	67 (17.0)	9 (9.6)	57 (20.1)	
100	27 (6.9)	7 (7.4)	18 (6.4)	
Unsure or don’t know	7 (1.8)	2 (2.1)	4 (1.4)	
In the past 12 mo, No. of unique patients treated with buprenorphine for an OUD				
No. of respondents	378	90	274	
0-10	45 (11.9)	15 (16.7)	28 (10.2)	<.001^a^
11-20	43 (11.4)	19 (21.1)	23 (8.4)	
20-50	109 (28.8)	25 (27.8)	78 (28.5)	
>50	181 (47.9)	31 (34.4)	145 (52.9)	
In the past 12 mo, No. of buprenorphine initiations done by respondent or supervisee				
No. of respondents	381	90	276	
0-10	72 (18.9)	24 (26.7)	44 (15.9)	.009^a^
11-20	87 (22.8)	25 (27.8)	54 (19.6)	
20-50	117 (30.7)	25 (27.8)	90 (32.6)	
>50	105 (27.6)	16 (17.8)	88 (31.9)	
In the past 12 mo, which of the following treatments have been prescribed or recommended for patients with OUD? (check all that apply)^b^				
No. of respondents	393	93	283	
Treatment for associated medical or psychiatric conditions	364 (91.9)	83 (89.2)	267 (94.3)	NA
Detoxification only	78 (19.7)	17 (18.3)	59 (20.8)	
Methadone maintenance treatment program	145 (36.6)	18 (19.4)	121 (42.8)	
Naltrexone or ER naltrexone	242 (61.1)	56 (60.2)	178 (62.9)	
Office-based buprenorphine treatment	342 (86.3)	71 (76.3)	256 (90.5)	
Abstinence-only treatment (no medication)	45 (11.4)	11 (11.8)	32 (11.3)	
Other	17 (4.3)	0 (0.0)	15 (5.3)	
Which of the following prescribed medications are available in your practice? (check all that apply)^b^				
No. of respondents	390	93	280	
Methadone	123 (31.5)	26 (28.0)	95 (33.9)	NA
Bunavail buccal film	46 (11.8)	17 (18.3)	26 (9.3)	
Cassipa sublingual film	7 (1.8)	2 (2.2)	2 (0.7)	
Suboxone sublingual tablet or film	377 (96.7)	89 (95.7)	273 (97.5)	
Subutex sublingual tablet	332 (85.1)	78 (83.9)	242 (86.4)	
Zubsolv sublingual tablets	223 (57.2)	53 (57.0)	162 (57.9)	
Lucemyra	46 (11.8)	11 (11.8)	35 (12.5)	
Buprenorphine treatment time to appointment at the primary location you prescribe from				
No. of respondents	381	91	276	
Patients can get appointments within 1-3 d	220 (57.7)	51 (56.0)	163 (59.1)	.67^a^
4-7 d	111 (29.1)	24 (26.4)	81 (29.3)	
8-30 d	35 (9.2)	12 (13.2)	21 (7.6)	
1-3 mo	3 (0.8)	1 (1.1)	2 (0.7)	
3-6 mo	1 (0.3)	0 (0.0)	1 (0.4)	
Unsure or don’t know	11 (2.9)	3 (3.3)	8 (2.9)	
In the past 12 mo, what percentage of your patients do you know or suspect were using fentanyl prior to initiating buprenorphine treatment?				
No. of respondents	396	94	285	
0	6 (1.5)	4 (4.3)	1 (0.4)	<.001^a^
≤25	72 (18.2)	24 (25.5)	43 (15.1)	
26-50	94 (23.7)	30 (31.9)	60 (21.1)	
51-75	76 (19.2)	19 (20.2)	52 (18.2)	
76-100	148 (37.4)	17 (18.1)	129 (45.3)	
When did you first notice that patients tested positive for fentanyl?				
No. of respondents	390	90	284	
Within the past 6 mo	42 (10.8)	14 (15.6)	28 (9.9)	<.001^a^
7-12 mo ago	46 (11.8)	14 (15.6)	31 (10.9)	
1-2 y ago	131 (33.6)	35 (38.9)	91 (32.0)	
≥3 y ago	146 (37.4)	17 (18.9)	122 (43.0)	
Unsure	25 (6.4)	10 (11.1)	12 (4.2)	
How do you know whether patients have recently used fentanyl? (check all that apply)^b^				
No. of respondents	395	93	285	
Patient self-report of fentanyl use	309 (78.2)	66 (71.0)	232 (81.4)	NA
Toxicology of biological samples	263 (66.6)	63 (67.7)	193 (67.7)	
Assume use based on community-level data on fentanyl use and availability	169 (42.8)	22 (23.7)	139 (48.8)	
Other	4 (1.0)	2 (2.2)	2 (0.7)	
Besides fentanyl and fentanyl analogs, which other drugs are complicating treatment? (check all that apply)^b^				
No. of respondents	392	91	284	
Methamphetamine or other illicit psychostimulants	342 (86.4)	81 (89.0)	248 (87.3)	NA
Xylazine	164 (41.4)	23 (25.3)	134 (47.2)	
Novel opioids (eg, etonitazepine)	49 (12.4)	7 (7.7)	40 (14.1)	
Designer or non-FDA approved benzodiazepines	126 (31.8)	24 (26.4)	100 (35.2)	
Synthetic cannabinoids (eg, K2/spice)	169 (42.7)	48 (52.7)	109 (38.4)	
Synthetic cathinones (eg, bath salts)	61 (15.4)	15 (16.5)	43 (15.1)	
Tianeptine (“gas station heroin”)	50 (12.6)	9 (9.9)	39 (13.7)	
Other	18 (4.6)	7 (7.7)	10 (3.5)	
Kratom	15 (3.8)	2 (2.2)	13 (4.6)	
Cocaine	9 (2.3)	2 (2.2)	7 (2.5)	
Alcohol	7 (1.8)	0 (0.0)	6 (2.1)	
Benzodiazepines	12 (3.0)	1 (1.1)	10 (3.5)	
What is the route of primary administration of fentanyl in the last 10 patients you inducted onto buprenorphine (check all that apply)^b^				
No. of respondents	394	93	284	
Injecting	250 (63.5)	59 (63.4)	180 (63.4)	NA
Smoking	205 (52.0)	45 (48.4)	152 (53.5)	
Sniffing	142 (36.0)	20 (21.5)	116 (40.8)	
Rectal insertion (boofing)	5 (1.3)	0 (0.0)	5 (1.8)	
Oral ingestion (parachuting)	100 (25.4)	29 (31.2)	66 (23.2)	
Unsure	23 (5.8)	9 (9.7)	11 (3.9)	
Other	3 (.8)	1 (1.1)	2 (0.7)	
How frequently did the last 10 patients you inducted onto buprenorphine use fentanyl prior to induction?				
No. of respondents	396	94	285	
Rarely (1-2 times per mo)	23 (5.8)	13 (13.8)	9 (3.2)	<.001^a^
Occasionally (1-2 times per wk)	80 (20.2)	28 (29.8)	48 (16.8)	
Frequently (5 times per wk)	99 (25.0)	29 (30.9)	64 (22.5)	
Daily or multiple times per day	194 (49.0)	24 (25.5)	164 (57.5)	

Abbreviations: ER, emergency room; FDA, US Food and Drug Administration; NA, not applicable; OUD, opioid use disorder.

^a^ *P* value from Pearson χ^2^ test.

^b^ The number of responses is larger than the number of respondents because the respondents were allowed to pick more than 1 answer. The percentages reported reflect the number of respondents checking the answer.

### Patients Using IMF

Among 390 respondents, 271 (69.5%) described patient reports of increased opioid withdrawal symptoms, 95 (24.4%) described patient reports of being “allergic” to BUP, and 84 (21.5%) described patient reports of a preference for methadone. Additionally, 189 (48.5%) reported that patients were dropping out or missing clinic appointments more frequently than those who were not using IMF (Table 3).

**Table 3.** Buprenorphine Initiation in Patients Using Fentanyl

Variable	Total sample, No. (%) (N = 396)	Reported ≥1 case of precipitated or prolonged withdrawal, No. (%) (n = 379)		*P* value
		No	Yes	
Have you encountered any of the following problems when trying to start patients who use fentanyl on buprenorphine? (check all that apply)^a^				
No. of respondents	390	90	284	
Increased opioid withdrawal symptoms per patient report	271 (69.5)	33 (36.7)	231 (81.3)	NA
More patients reporting being “allergic” to buprenorphine	95 (24.4)	14 (15.6)	78 (27.5)	
More patients reporting a preference for methadone treatment	84 (21.5)	7 (7.8)	73 (25.7)	
Patients more frequently dropping out after first appointment or increasing No. of patients missing appointments	189 (48.5)	33 (36.7)	153 (53.9)	
Other problems	37 (9.5)	7 (7.8)	28 (9.9)	
I have not encountered any problems	39 (10.0)	25 (27.8)	11 (3.9)	
Have you had to increase the number of buprenorphine initiation attempts among patients using fentanyl?				
No. of respondents	224	32	181	
Never	10 (4.4)	4 (12.5)	4 (2.2)	<.001^b^
Rarely	22 (9.8)	7 (21.9)	13 (7.2)	
Sometimes	75 (33.5)	10 (31.2)	63 (34.8)	
Occasionally	77 (34.4)	6 (18.8)	70 (38.7)	
Always	19 (8.5)	0	19 (10.5)	
Not sure	21 (9.4)	5 (15.6)	12 (6.6)	
In the past year, what percentage patients have you seen who were using fentanyl and preferred another treatment option other than buprenorphine?				
No. of respondents	225	33	181	
≤10	70 (31.1)	16 (48.5)	48 (26.5)	<.001^c^
11-49	117 (52.0)	9 (27.3)	106 (58.6)	
50-79	24 (10.7)	2 (6.1)	22 (12.2)	
80-99	5 (2.2)	2 (6.1)	3 (1.7)	
Unsure	9 (4.0)	4 (12.1)	2 (1.1)	
In the past year, have any of your patients reported or experienced precipitated withdrawal (an increase >10 points on the COWS scale or a rapid onset of new withdrawal symptoms after buprenorphine administration) when being inducted onto buprenorphine?				
No. of respondents	394	94	284	
No–not aware of any patients experiencing precipitated withdrawal	136 (34.5)	94 (100)	37 (13.0)	<.001^b^
Yes–many patients experience precipitated withdrawal whether or not they have used fentanyl	94 (23.9)	0	94 (33.1)	
Yes–I am primarily seeing precipitated withdrawal among patients who are using fentanyl	148 (37.6)	0	148 (52.1)	
Unsure	16 (4.1)	0	5 (1.8)	
In the past year, have any of your patients described prolonged withdrawal (symptoms lasting more than 72 h) when being inducted onto buprenorphine?				
No. of respondents	392	94	284	
No–not aware of any patients experiencing prolonged withdrawal	162 (41.3)	94 (100)	63 (22.2)	<.001^b^
Yes–many patients experience prolonged withdrawal whether or not they have used fentanyl	63 (16.1)	0	63 (22.2)	
Yes–but only or mostly my patients using fentanyl experience prolonged withdrawal	144 (36.7)	0	144 (50.7)	
Unsure or do not know	23 (5.9)	0	14 (4.9)	
How important of a problem do you think that precipitated or prolonged opioid withdrawal is when starting buprenorphine treatment among patients using fentanyl?				
No. of respondents	393	94	282	
Not at all important	18 (4.6)	13 (13.8)	4 (1.4)	<.001^c^
Somewhat important	120 (30.5)	42 (44.7)	71 (25.2)	
Moderately important	97 (24.7)	18 (19.1)	75 (26.6)	
Very important	158 (40.2)	21 (22.3)	132 (46.8)	
What other prescription and over-the-counter medications do you prescribe, dispense, or recommend to your patients to use for opioid withdrawal within the past year? (check all that apply)^a^				
No. of respondents	392	91	284	
Diphenhydramine	125 (31.9)	38 (41.8)	82 (28.9)	NA
Ibuprofen	210 (53.6)	44 (48.4)	157 (55.3)	
Dicyclomine	141 (36.0)	19 (20.9)	117 (41.2)	
Ondansetron	319 (81.4)	68 (74.7)	238 (83.8)	
Acetaminophen	176 (44.9)	36 (39.6)	135 (47.5)	
Gabapentin	157 (40.1)	34 (37.4)	115 (40.5)	
Clonidine	344 (87.8)	73 (80.2)	255 (89.8)	
Phenobarbital	8 (2.0)	1 (1.1)	7 (2.5)	
Other	92 (23.5)	7 (7.7)	82 (28.9)	
Approximately what percentage of patients whom you treat for opioid use disorder do you refer to methadone treatment after unable to achieve therapeutic dose?				
No. of respondents	392	93	282	
None	130 (33.2)	43 (46.2)	81 (28.7)	.051^c^
≤25	158 (40.3)	31 (33.3)	120 (42.6)	
26-50	33 (8.4)	6 (6.5)	27 (9.6)	
51-75	14 (3.6)	3 (3.2)	11 (3.9)	
76-100	27 (6.9)	3 (3.2)	23 (8.2)	
Unsure	30 (7.7)	7 (7.5)	20 (7.1)	

Abbreviations: COWS, Clinical Opioid Withdrawal Scale; NA, not applicable.

^a^ The number of responses is larger than the number of respondents because the respondents were allowed to pick more than 1 answer. The percentages reported reflect the number of respondents checking the answer.

^b^ *P *value from Fisher exact.

^c^ *P* value from Pearson χ^2^ test.

### Buprenorphine Induction Problems

Nearly three-quarters of participants (284 of 390 [72.8%]) reported having encountered problems (either precipitated and/or prolonged withdrawal) when initiating patients using IMF onto BUP in the past year, with 242 of 394 (61.4%) reporting at least 1 episode of precipitated withdrawal and 207 of 392 (52.8%) reporting prolonged withdrawal. When asked how important of a problem precipitated or prolonged withdrawal was, 18 of 393 (4.6%) stated it was not important at all, whereas 255 of 393 (64.9%) found it moderately or very important (Table 3). A majority (244 of 284 [85.9]) reported first noticing problems with initiating BUP within the past 6 months. Of 392 participants, 146 (37.2%) reported that all their patients were eventually able to achieve a therapeutic dose of BUP, whereas 41 (10.5%) reported that more than half of their patients were unable and were subsequently referred to methadone treatment (Table 3).

Results from multivariable logistic regression modeling indicated that doctors, physician assistants, and nurse practitioners waivered to treat more than 100 patients were more likely to report problems initiating BUP than those waivered to treat fewer patients (OR, 1.92; 95% CI, 1.08-3.40). Additionally, clinicians with at least 75% of their patients using IMF were more likely to report problems initiating BUP than clinicians who reported no patients using IMF (OR, 6.31; 95% CI, 2.59-15.35). Clinicians reported that patients inducted in settings other than inpatient were more likely to experience problems initiating BUP (OR, 2.79; 95% CI, 1.39-5.61). Geographic region, practice setting, and the number of years the clinician had practiced were not associated with problems initiating BUP.

### Strategies to Resolve BUP Initiation Problems

More than half of participants (264 of 392 [67.3%]) reported they had modified their standard BUP treatment protocols, which included changing medication initiation protocols (56 [14.3%]), changing how they counseled patients (46 [11.7%]), or changing both medication and counseling protocols (162 [41.3%]). Medication protocol changes, reported by 218 respondents, included prescribing comfort medications (149 [68.3%]), waiting longer to start BUP (116 [53.2%]), changing the first dose of BUP (148 [67.9%]), and changing the titration schedule to lower or higher dosing (167 [76.6%]) (Table 4). Of 395 respondents, 75 (19.0%) reported initiating BUP at less than 2 mg, 72 (18.2%) reported initiating BUP at 2 mg, and 45 (11.4%) reported initiating BUP at 6 to 24 mg. Of 396 respondents, 122 (30.8%) reported encountering pharmacy-related challenges that affected their modified BUP induction practices, including issues such as BUP dosage not covered by the patient’s insurance or low dose not available in community-based pharmacies.

**Table 4.** Modification of Buprenorphine Initiation

Variable	Total sample, No. (%) (N = 396)	Reported ≥1 case of precipitated or prolonged withdrawal, No. (%) (n = 379)^a^		*P* value
		No	Yes	
In general, have you modified how you initiate buprenorphine treatment in patients using fentanyl?				
No. of respondents	392	94	281	
No	120 (30.6)	52 (55.3)	59 (21.0)	<.001^b^
Yes: different medications and/or different protocols are followed	56 (14.3)	11 (11.7)	42 (14.9)	
Yes: we have changed how we counsel patients	46 (11.7)	9 (9.6)	36 (12.8)	
Yes: we have changed both medication protocols and how we counsel patients	162 (41.3)	18 (19.1)	141 (50.2)	
Unsure	8 (2.0)	4 (4.3)	3 (1.1)	
What modifications to the usual buprenorphine initiation protocol made at your clinic site for patients using fentanyl have you made? (check all that apply)^c^				
No. of respondents	218	29	183	
Comfort medications prescribed	149 (68.3)	19 (65.5)	128 (69.9)	NA
Longer lag time before first dose of buprenorphine	116 (53.2)	13 (44.8)	100 (54.6)	
Change in dosage of first dose of buprenorphine	148 (67.9)	13 (44.8)	131 (71.6)	
Change in titration schedule for buprenorphine (low or high dose)	167 (76.6)	18 (62.1)	146 (79.8)	
Other	5 (2.3)	0 (0.0)	5 (2.7)	
What do you think is needed to optimize addiction treatment engagement and retention in patients using fentanyl? (check all that apply)^c^				
No. of respondents	393	94	282	
Inpatient detox	207 (52.7)	48 (51.1)	149 (52.8)	NA
Methadone treatment	87 (22.1)	9 (9.6)	74 (26.2)	
Alternative buprenorphine dosing protocols	246 (62.6)	45 (47.9)	189 (67.0)	
Additional medications to manage opioid withdrawal	251 (63.9)	52 (55.3)	191 (67.7)	
Using short-acting agonists as adjunctive to treatment	138 (35.1)	19 (20.2)	115 (40.8)	
Other	28 (7.1)	10 (10.6)	16 (5.7)	

Abbreviation: NA, not applicable.

^a^ Sample size does not equal 396 for the analytic sample because only those that answered yes to the primary outcome questions (buprenorphine induction problems) were counted.

^b^ *P* value from Pearson χ^2^ test.

^c^ The number of responses is larger than the number of respondents because the respondents were allowed to pick more than 1 answer. The percentages reported reflect the number of respondents checking the answer.

## Discussion

This is, to our knowledge, the first national study of clinician-reported problems related to initiating BUP among patients using IMF. Previous studies, largely conducted in ED settings, have reported a very low incidence rate of precipitated opioid withdrawal (<1%) when starting BUP treatment in patients using IMF.^20^ That finding is contrary to anecdotal reports and to retrospective studies from clinicians across the country,^13,37^ which suggest that precipitated withdrawal may exceed 16%. The present study did not yield directly comparable data because the unit of analysis was clinicians, not patients. However, 72% of clinicians we surveyed reported seeing both precipitated and/or prolonged withdrawal in patients using IMF in the past year, and nearly all reported that difficulty initiating patients onto BUP was an important problem.

The variability in the rate of precipitated withdrawal may be due to several factors, including the clinical setting in which BUP induction occurs and the type of patients seen. Significant individual-level differences in fentanyl clearance occur, with some patients continuing to test positive for fentanyl up to 13 days after cessation.^8^ Unfortunately, pharmacokinetic studies^7,8^ have provided limited insights, as these studies used pharmaceutical-grade fentanyl, which may not generalize to IMF, and their samples were composed of healthy individuals rather than those chronically using IMF. Existing studies have found that older individuals and those with overweight are slower to metabolize fentanyl^15,38^; it is also possible that co-use of IMF with other drugs, particularly those metabolized through CYP3A, may slow clearance.^39^ Additionally, fentanyl can have multiple peak levels or a rebound effect, possibly explaining why presentation of precipitated withdrawal may vary by clinical settings.^15,38^

Undoubtedly, the discrepancies of reported precipitated opioid withdrawal are due to its measurement and lack of objective psychometrically validated instruments. We suspect that clinicians modified their BUP induction protocols due to significant clinical challenges, irrespective of whether precipitated or prolonged opioid withdrawal was objectively assessed or subjectively reported by patients. Notably, problems initiating BUP treatment were not geographically isolated; rather, they appear to be ubiquitous in this study.

Additionally, polysubstance use, including stimulants such as methamphetamines and novel psychoactive substances, is prolific^39,40,41,42,43^ and may complicate BUP initiation.^44^ Lags in epidemiological data and changing trends in drug use among patients seeking treatment make it difficult for clinicians to ascertain the potential influence of specific drugs on treatment engagement and retention. Current drug surveillance efforts could be expanded to include clinician reports of problems initiating drug treatment and be triangulated with objective drug toxicology data to provide a more comprehensive perspective and improve early identification of clinically relevant new drug trends.

### Limitations

This study has the following limitations. We examined clinicians’ subjective reports of BUP initiation problems and strategies used to remedy problems attributed to IMF. Precipitated withdrawal and prolonged withdrawal lack standard definitions across research studies and clinical practice. Participants were asked to recall clinical encounters over the past year, which may lend to recall bias. Moreover, clinicians who experienced more BUP induction problems may have been overrepresented among participants. In this survey, the data reflects participants’ professional opinions, not objective measures of patient-level precipitated withdrawal. The 26.1% response rate is similar to or better than other surveys conducted with this population.^45^ Additionally, the different initial and maintenance doses of BUP measured may not reflect a more complex strategy to mitigate opioid withdrawal, including the timing of different doses, at different points of patient experience of withdrawal, or how long it takes to achieve maintenance dosing or abstinent illicit drug use within and among different institutions and treatment milieus.

## Conclusions

In this survey study, clinicians across the country reported problems initiating BUP treatment among patients using IMF, and while in most cases this refers to patients experiencing precipitated and/or prolonged withdrawal, it includes other problems, such as patients’ reports that BUP does not work for them or patient preferences for methadone treatment. Despite the ubiquitous problems reported that are rated seriously by prescribers and prompting changes in practice, BUP remains a life-saving treatment for OUD. Our findings should not in any way be interpreted to discourage BUP initiations or use in this population. It is unclear how adjusted induction methods influence BUP engagement and retention rates, which is a topic for future study.

## Notes

Supplement.
Data Sharing Statement
