# Supplementary material for: Barriers to Buprenorphine Initiation in Patients Using Fentanyl
Source: JAMA Netw Open. 2026 Jan 5;9(1):e2552136. doi: 10.1001/jamanetworkopen.2025.52136 (PMC12771233; doi:10.1001/jamanetworkopen.2025.52136)
Supplement: Supplement. — Data Sharing Statement [file jamanetwopen-e2552136-s001.pdf]

Data Sharing Statement

Kawasaki. Barriers to Buprenorphine Initiation in Patients Using Fentanyl. *JAMA Netw Open*. Published January 05, 2026. doi:10.1001/jamanetworkopen.2025.52136

Data
Data available: Yes
Data types: Deidentified participant data
How to access data: The data will be available on the NIDA Data Share website. https://datashare.nida.nih.gov/
When available: beginning date: 06-01-2026

Supporting Documents
Document types: Statistical/analytic code, Informed consent form
How to access documents: Anyone requesting the data that agrees to the data use terms
When available: beginning date: 06-01-2026

Additional Information
Who can access the data: Anyone requesting the data that agrees to the data use terms
Types of analyses: The NIDA Data Share makes the de-indentified data available and supporting documentation. Other materials, including the study protocol, may be available on the NIDA CTN Dissemination Library https://ctnlibrary.org
Mechanisms of data availability: The NIDA Data Share is a public database with accessibility outlined in the Registration Agreement and the Data Share Policy.
